# Detection of *Plasmodium knowlesi* DNA in the urine and faeces of a Japanese macaque (*Macaca fuscata*) over the course of an experimentally induced infection

**DOI:** 10.1186/1475-2875-13-373

**Published:** 2014-09-19

**Authors:** Satoru Kawai, Megumi Sato, Naoko Kato-Hayashi, Hisashi Kishi, Michael A Huffman, Yoshimasa Maeno, Richard Culleton, Shusuke Nakazawa

**Affiliations:** Laboratory of Tropical Medicine and Parasitology, Dokkyo Medical University, Mibu, Tochigi, 321-0293 Japan; Graduate School of Health Sciences, Niigata University, Niigata, Japan; Laboratory of Hygiene, Dokkyo Medical University, Mibu, Tochigi, Japan; Section of Social Systems Evolution, Primate Research Institute, Kyoto University, Inuyama, Aichi, Japan; Department of Virology and Parasitology, Fujita Health University School of Medicine, Toyoake, Aichi, Japan; Malaria Unit, Institute of Tropical Medicine, Nagasaki University, Nagasaki, Japan; Department of Protozoology, Institute of Tropical Medicine, Nagasaki University, Nagasaki, Japan

**Keywords:** *Plasmodium knowlesi*, Zoonotic malaria, Japanese macaque, Urine, Faeces, Nested PCR, Cytochrome *b*, Chloroquine sulphate

## Abstract

**Background:**

Diagnostic techniques based on PCR for the detection of *Plasmodium* DNA can be highly sensitive and specific. The vast majority of these techniques rely, however, on the invasive sampling of blood from infected hosts. There is, currently, considerable interest in the possibility of using body fluids other than blood as sources of parasite DNA for PCR diagnosis.

**Methods:**

Urine and faeces were obtained from a *Plasmodium knowlesi* infected-Japanese macaque (*Macaca fuscata*) over the course of an experimentally induced infection. *P. knowlesi* DNA (*Pk*DNA) extracted from urine and faeces were monitored by nested PCR targeting the *P. knowlesi* specific cytochrome *b* (*cytb*) gene.

**Results:**

Urinary *Pk*DNA was detected on day 2, but was not amplified using DNA templates extracted from the samples on day 4, day 5 and day 6. Subsequently, urinary *Pk*DNA was detected from day 7 until day 11, and from day 20 until day 30. *Pk*DNA in faeces was detected from day 7 until day 11, and from day 20 until day 37. Moreover, real-time quantitative PCR showed a remarkable increase in the amount of urinary *Pk*DNA following anti-malarial treatment. This might have been due to the release of a large amount of *Pk*DNA from the degraded parasites as a result of the anti-malarial treatment, leading to excretion of *Pk*DNA in the urine.

**Conclusions:**

The *cytb*-PCR system using urine and faecal samples is of potential use in molecular epidemiological surveys of malaria. In particular, monkey faecal samples could be useful for the detection of zoonotic primate malaria in its natural hosts.

**Electronic supplementary material:**

The online version of this article (doi:10.1186/1475-2875-13-373) contains supplementary material, which is available to authorized users.

## Background

To date, microscopic examination of Giemsa-stained blood smears remains the most common and most trusted technique for the detection of malaria parasites. There are many alternative methods for parasite detection, each with their own strengths and weaknesses compared to microscopy. These include various techniques, such as rapid immuno-chromatographic tests that detect circulating malaria antigens or antibodies; and polymerase chain reaction (PCR)-based assays for the detection of parasite DNA in peripheral blood [1]. Although these techniques are now available for diagnosing malaria, current approaches rely on the drawing of blood by finger pricking or venipuncture. The requirement for repeated drawing of blood samples for longitudinal follow-up studies or continuous monitoring in the case of vaccine efficacy tests may at times result in poor compliance, especially among infants, young children and pregnant women [2]. In addition, the procedure for drawing blood, if not carried out under stringent conditions, is associated with the risk of acquiring needle-borne infections, such as hepatitis B virus and human immunodeficiency virus [3]. Therefore, the development of noninvasive approaches using body fluids other than blood would improve malaria diagnosis and the completion of epidemiological surveys. It is also likely that noninvasive approaches to surveillance would improve population coverage.

*Plasmodium knowlesi* is a simian malaria parasite the natural hosts of which long-tailed macaques (*Macaca fascicularis*), pig-tailed macaques (*Macaca nemestrina*), and banded leaf monkeys (*Presbytis malalophos*), all of which inhabit large areas of Southeast Asia [4]. Following the report in 2004 of a large focus of human infections with *P. knowlesi* in Peninsular Malaysia, zoonotic *P. knowlesi* infections were described from numerous countries in Southeast Asia [4, 5]. Human *P. knowlesi* infection may often have been misidentified by microscopy as *Plasmodium malariae* or *Plasmodium falciparum* due to morphologic similarities, leading to underestimations of its true prevalence [4]. Thus, PCR-based assays and sequence analysis are probably the most reliable methods of identifying *P. knowlesi* infection. Wide-scale surveys based on PCR are required to assess the prevalence and distribution of the reservoir hosts of zoonotic primate malaria. To date, epidemiological surveys involving the natural hosts of *P. knowlesi* rely on the detection of parasite DNA extracted from fresh, frozen or dried blood obtained from wild monkeys [6, 7]. As invasive sampling of non-human primates is problematic both practically and ethically, non-invasive sampling methods are desirable.

Recent studies have shown that the saliva, urine and faeces of malaria patients contain trace amounts of *Plasmodium* DNA that is amplifiable by PCR and, therefore, could be used as an alternative source of specimens for epidemiological surveys [8–13]. Detection of parasite DNA fragments in urine by PCR has also been employed in the diagnosis of various other parasitic diseases, such as those caused by *Toxoplasma gondii*, *Leishmania* spp., *Trichomonas vaginalis*, *Entamoeba histolytica*, and *Schistosoma mansoni*, although at present it is still not widely used [14–18]. Despite the lower sensitivity of PCR for the detection of parasite DNA from urine and faeces compared to blood, noninvasive sampling may be preferable due to ease of use. Moreover, if parasite DNA can be detected using the excreta of free-living monkeys, it will provide a useful tool for epidemiological surveys on zoonotic primate malaria. Here, the detection of *P. knowlesi* DNA (*Pk*DNA) in urine and faeces obtained from a Japanese macaque (*Macaca fuscata*) over the course of an experimentally induced infection is demonstrated. The relationship between parasitaemia and the detection of *Pk*DNA in both faeces and urine is analysed, and the diagnostic performance of *Pk*DNA detection is assessed.

## Methods

### Animals and infection procedure

A four-year-old 5.0 kg female Japanese macaque (*Macacca fuscata*), a second-generation offspring bred in captivity that had never been infected with malaria parasites, was used for this study. The animal was kept in an individual cage in a controlled environment at 25-27°C and 30-60% humidity, and given commercial food pellets supplemented with fresh fruits. Throughout the course of the experiment, investigators adhered to the Guidelines for the Use of Experimental Animals authorized by the Japanese Association for Laboratory Animal Science. The protocol was approved by the Committee on the Ethics of Animal Experiments of Dokkyo Medical University (Permit Number: 0656). The monkey was inoculated intravenously with 1×10^9^ frozen *P. knowlesi* H strain (ATCC No. 30158) parasitized red blood cells (PRBCs) obtained from another infected Japanese macaque. Thin blood films were prepared from peripheral blood obtained through earpick at day 0, 2, 4, 5, 6, 7, 8, 9, 11, 15, 20, 21, 22, 23, 25, 30, 37, 40, 50, and 60. Following Giemsa staining, parasitaemia was counted in a total 10^4^ erythrocytes with an optical microscope. Heparinized blood samples for DNA extraction and hematological examinations were obtained intravenously from the infected monkey on day 0, 2, 4, 6, 7, 9, 11, 15, 20, 25, 30, 37, 40, 50, and 60. Whole blood samples for DNA extraction were stored at −80°C until DNA extraction.

### Sampling procedures for urine and faeces

Urine and faecal samples for DNA extraction were obtained from the infected monkey on days 0, 2, 4, 5, 6, 7, 8, 9, 10, 11, 12, 15, 20, 21, 22, 25, 30, 37, 40, 50, and 60. A sampling net and tray were set under the monkey cage for three hours in the morning (9:00 AM-12:00 PM) from which fresh urine and faeces were collected from the infected monkey (Additional file 1). They were replaced and cleaned thoroughly after every sampling to avoid contamination. Negative control samples were obtained from two non-infected monkeys (ID: J83 and J84) according to the same procedures. One gram of fresh faeces was mixed with 2.0 mL RNAlater® solution (Ambion®, Austin, TX), and the faecal and urine samples were promptly stored at −80°C until DNA extraction. After thawing the urine samples, haemoglobin concentrations were measured using a commercial kit (Wako Pure Chemical Industries, Ltd., Osaka Japan). Bleeding due to menstruation was not observed during the experimental period.

### Anti-malarial treatment

**Figure 1:**
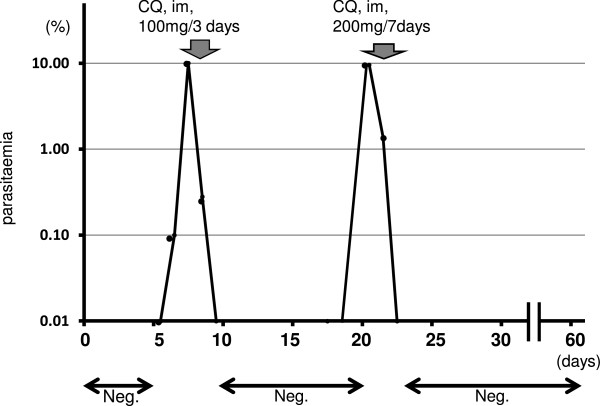
**Parasitaemia during the course of infection.** The arrows indicate chloroquine treatment. : Parasite negative in peripheral blood by microscopy.

On the 7th day post-infection, an injectable solution of chloroquine sulphate 40 mg (NIVAQUINE™; Bangladesh Pharmaceutical Industry Lit, Dhaka, Bangladesh) was administered intramuscularly, followed by 20 mg at 6 hr, 24 hr and 48 hr after initial administration for a total of 100 mg (Figure 1). Parasitaemia dropped to sub-microscopic levels from day 9 post-infection (two days following first drug treatment), but recrudesced at day 20 post-infection. On day 20 post-infection, 40 mg of NIVAQUINE™ was administered, followed by 20 mg at 6 hr, 12 hr, 24 hr and 48 hr after first administration. On days 23, 24, 25 and 26, 20 mg of NIVAQUINE™ was administered to prevent recrudescence of the parasite (Figure 1).

### DNA extraction steps

DNA was extracted from 100 μL of each whole blood sample by using the Illustra™ blood genomicPrep Mini Spin Kit (GE Healthcare, Buckinghamshire, UK) according to the manufacturer’s instructions, and then eluted into 200 μL of elution buffer for the kit. DNA extraction from urine samples was performed with a QIAamp® Viral RNA Mini Kit (Qiagen, Hilden, Germany) according to the manufacturer’s instructions. Briefly, 4 mL of urine sample was applied to the centrifugal filter units (Amicon Ultra-15, Millipore Ireland B.V., County Cork, Republic of Ireland), followed by centrifugation for 10 minutes at 2,000× *g*. 200 μL of the concentrated samples were applied to QIAam® spin columns and centrifuged. After two washes, DNA was eluted with 80 μL of the supplied buffer at room temperature. DNA extraction from faecal samples (1 g of faeces/2 ml RNAlate®) was performed with QIAamp® DNA Stool Mini Kit (Qiagen) according to the manufacturer’s instructions, and then eluted into 50 μL of elution buffer for the kit. DNA extractions from each sample were performed within one month of the sampling, and the purity and concentration of DNA samples were determined using NanoDrop apparatus (Thermo Fisher Scientific, Waltham, MA). DNA was stored at −20°C until use.

### Nested PCR for amplification of *Pk*DNA

Nested PCR (nPCR) was performed using a TaKaRa PCR Thermal Cycler Dice (TAKARA BIO INC. Shiga, Japan). Detection of *Pk*DNA in blood, urine and faeces was performed by nPCR targeting the mitochondrial cytochrome *b* gene (*cytb*). Primers for the primary PCR were *Plasmodium* genus-specific and those for secondary PCR were specific for *P. knowlesi*. Forward primers for the primary and secondary PCRs were as reported by Putaporntip *et al*. and the reverse primers were as reported by Tanizaki *et al*. [12, 19]. DNA amplification was carried out in a total volume of 20 μL containing 1 μL of DNA template, 10 μL of 2X PCR buffer (AmpliTaq Gold PCR Master Mix, AB Applied Biosystems, Branchburg, NJ, USA), and 0.3 μM of each primer. Final volume of original samples per each PCR was as follows; blood was 0.5 μL; urine was 50 μL; faeces was 50 mg. Primary amplification conditions were as follows: initial denaturation at 95°C for 5 min, followed by 35 cycles (94°C for 40 sec, 50°C for 30 sec and 72°C for 30 sec) and final extension at 72°C for 4 min. The product was diluted 1:50 in DEPC treated water (Invitrogen, Carlsbad, CA, USA), and 2 μL of the diluted product was subsequently subjected to secondary amplification. The concentration of the primers and other constituents for secondary amplification was identical to those of the primary amplification. Secondary amplification conditions consisted of 25 cycles under the same conditions as the primary PCR, followed by a final extension at 72°C for 4 min. A total of 10 μL of PCR products were visualized by gel electrophoresis on a 2% agarose gel.

### Real-time quantitative PCR

To measure the amount of *Pk*DNA in the urine after anti-malarial treatment, quantitative nested real-time PCR (qPCR) was carried out. The qPCR in this study was designed referring to a previous report on diagnosis of Barmah forest virus infection by a nested real-time SYBR green RT-PCR assay [20]. Urine samples for qPCR were collected from the monkey prior to and following administration of the first and second anti-malarial treatment (Figure 1). The first set of urine samples was collected on day 5, day 6, 10:00 on day 7, 16:00 on day 7, 10:00 on day 8 and on day 10, and the second set of urine samples was collected on day 19, 10:30 on day 20, 16:30 on day 20, 10:30 on day 21, day 22 and day 23. qPCR was conducted using the first PCR products obtained from genus specific PCR used as a DNA template. All samples were tested in triplicate. The PCR mix consisted of 12.5 μL SYBR® *Premix Ex Taq*™ II, Tli RNaseH Plus (TAKARA BIO INC.), 0.4 μM of each primer (e.g., PkCBF and PkCBR-ed) [12, 19], and 2 μL of template DNA in a 25 μL final reaction mix. PCR was performed in a Thermal Cycler Dice®, Real Time System II (TAKARA BIO INC.) under the following conditions: 30 seconds at 95°C for initial denaturation, followed by 40 cycles (95°C for 5 sec and 50°C for 30 sec) with melting curve analysis (95°C for 15 sec, 60°C for 30 sec and 95°C for 15 sec).

### Standard curve for qPCR

To produce the standard curve for qPCR, genomic DNA of *P. knowlesi* H strain was prepared from infected blood obtained from another *P. knowlesi*-infected monkey (ID: J60). Parasitaemia in the blood was determined as 27%. DNA extraction from infected blood was performed with the Illustra™ blood genomicPrep Mini Spin Kit according to the manufacturer’s instructions. Final concentration of DNA dissolved in 200 μL TE buffer was 57.6 ng/μL. The DNA solution was diluted 1:1,000 with TE buffer, which was defined as 10^4^ “Plasmodial units (P units)”. The standard curve for *Pk*DNA was obtained from five serial dilutions (57.6 ng/μL ×1, 57.6 ng/μL ×10^−1^, 57.6 ng/μL ×10^−2^, 57.6 ng/μL ×10^−3^ and 57.6 ng/μL ×10^−4^). Two negative controls consisted of sterile water. Primers for the first PCR were used PCBF and PCBR-ed [12, 19]. The first PCR was carried out in a total volume of 20 μL containing 1 μL of DNA template, 10 μL of 2X PCR buffer (AmpliTaq Gold PCR Master Mix), and 0.3 μM of each primer. Primary amplification conditions were as follows: initial denaturation at 95°C for 5 min, followed by 35 cycles (94°C for 40 sec, 50°C for 30 sec and 72°C for 30 sec) and final extension at 72°C for 4 min. The first PCR product was diluted 1:50 in DEPC treated water (Invitrogen), and 2 μL of the diluted product was subsequently subjected to qPCR for the standard curve. The PCR mix consisted of 12.5 μL SYBR® *Premix Ex Taq*™ II, Tli RNaseH Plus (TAKARA BIO INC.), 0.4 μM of each primer (e.g., PkCBF and PkCBR-ed) [12, 19], and 2 μL of template DNA in a 25 μL final reaction mix. Secondary PCR for the standard curve was performed same condition as the sample analysis. Results of the qPCR are expressed in terms of “P units”.

## Results

### Parasitaemia and clinical examinations

Parasites were first detected in the peripheral blood by microscopy on day 5 post infection, and, thereafter, parasitaemia increased sharply to around 10% on day 7 (Figure 1). On day 7, parasitaemia decreased markedly after initial treatment with CQ, and no parasites were detectable in the peripheral blood by microscopy from day 9 to day 15 post-infection. However, on day 20, parasitaemia rose to 9.53%, and the monkey was administered anti-malarial drug once again (Figure 1). Parasitaemia decreased remarkably after this second treatment, with no second recrudescence observed for the remainder of the experiment. For this experiment, we monitored the kinetics of blood creatinine (CREA) and blood urea nitrogen (BUN) levels as an indicator of renal function (Table 1). On day 7, CREA and BUN levels experienced temporarily mild elevations as parasitaemia reached its primary peak; thereafter, levels ranged from 0.46 to 0.62 mg/dL (mean ± SD: 0.55 ± 0.04 mg/dL) and 10.3 to 18.8 mg/dL (15.1 ± 2.64), respectively. These concentrations were within the normal range of CREA (0.81 ± 0.28 mg/dL) and BUN (16.8 ± 5.2 mg/dL) in Japanese macaques [21]. In addition to blood haemoglobin levels, we monitored the kinetics of urinary haemoglobin levels (Table 1). On day 7 and 20, haemoglobin levels in the urine were elevated, correlating with increase in parasitaemia and with anti-malarial treatment. Urine samples that contained high haemoglobin concentrations showed a reddish colour.

**Table 1:** **Results of clinical examination over the course of the experiment**

Day after infection															
		0	4	6	7	9	11	15	20	25	30	37	40	50	60
Para.	%	-	-	0.1	10.08	-	-	-	9.58	-	-	-	-	-	-
WBC	×10^3^/μL	5.38	7.04	8.01	5.09	7.61	6.23	5.34	5.80	10.42	4.57	4.19	5.44	5.42	5.84
RBC	×10^4^/μL	512	471	481	403	329	302	327	312	258	327	401	403	452	428
HGB	g/dL	14.2	13.2	13.4	11.5	8.9	8.2	9.1	8.9	7.0	9.3	11.3	11.3	12.5	11.9
HCT	%	44.4	40.8	41.7	36.6	29.5	26.8	29.2	29.6	24.8	31.1	37.7	37.3	39.1	37.4
MCV	FI	86.7	86.6	86.7	90.8	89.7	88.7	89.3	94.9	96.1	95.1	94.0	92.6	86.5	87.4
MCH	Pg	27.7	28.0	27.9	28.5	27.1	27.2	27.8	28.5	27.1	28.4	28.2	28.0	27.7	27.8
MCHC	%	32.0	32.4	32.1	31.4	30.2	30.6	31.2	30.1	28.2	29.9	30.0	30.3	32.0	31.8
ALB	g/dL	4.7	4.4	4.4	3.5	3.3	3.3	3.6	3.1	3.1	3.2	3.9	3.8	4.1	3.9
AST	IU/L	27	24	27	51	42	31	24	52	50	25	23	28	30	19
ALT	IU/L	52	40	44	42	47	39	509	25	40	31	27	27	32	23
LD	IU/L	360	342	342	638	711	611	959	882	1277	533	302	569	489	265
ALP	IU/L	790	892	909	704	973	1113	73	932	859	723	690	722	647	640
γ -GTP	IU/L	96	84	88	68	70	63	0.51	69	67	72	84	81	90	82
Cr	mg/dL	00	0.69	0.66	0.82	0.53	0.46	14.3	0.62	0.54	0.53	0.55	0.58	0.59	0.59
BUN	mg/dL	0.67	15.4	16.9	22.2	10.3	11.2	0.1	13.6	16.9	15.1	16.2	18.0	16.6	18.8
BIL	mg/dL	13.9	0.1	0.1	0.2	0.1	0.2	0.01	0.05	0.1	0.1	0.1	0.1	0.1	0.1
BRP	mg/dL	0.01	<0.01	0.03	7.89	6.26	0.25	0.01	10.40	0.24	0.01	0.01	0.01	<0.01	<0.01
U-HGB^*^	mg/dL	56.8	57.9	58.4	102.0	65.0	65.0	68.3	213.6	62.8	54.5	54.0	60.1	52.8	52.8

U-HG^*:^ Urinary hemoglobin level.

### Detection of *Pk*DNA from blood, urine and faeces

**Figure 2:**
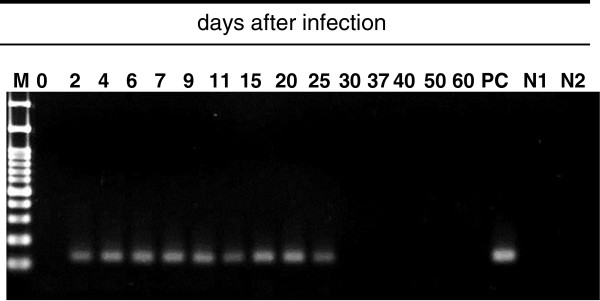
**Detection of** ***Pk*** **DNA in peripheral blood over the course of the experiment.** PC: positive control, N1: negative control for 1st PCR, N2: negative control for 2nd PCR.

**Figure 3:**
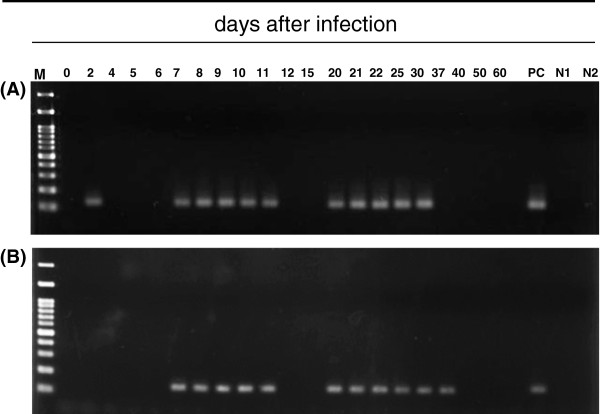
**Detection of** ***Pk*** **DNA in urine (A) and faecal samples (B) over the course of the experiment.** PC: positive control, N1: negative control for 1st PCR, N2: negative control for 2nd PCR.

The infection course was monitored by nPCR using DNA templates obtained from whole blood, urine and faeces. *Pk*DNA was first detected in whole blood on day 2, where it remained detectable until day 25 post-infection (Figure 2). Urinary *Pk*DNA was detected initially on day 2, but was not detected on day 4, day 5 and day 6 (Figure 3A). Subsequently, *Pk*DNA in urine was detected from day 7 until day 11, and from day 20 until day 30. Additionally, *Pk*DNA in the faeces was detected from day 7 until day 11, and from day 20 until day 37 (Figure 3B). The negative control samples obtained from two non-infected monkeys showed negative reaction.

### Real-time quantitative PCR

**Figure 4:**
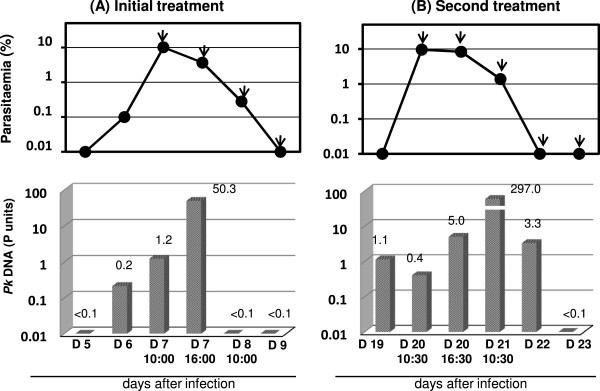
**Kinetics of** ***Pk*** **DNA concentration in urine samples. (A)**: Initial treatment, **(B)**: Second treatment. The arrows indicate intramuscular administration of chloroquine.

The standard curve constructed using five serial dilutions of the DNA solution showed good linearity with a consistent coefficient (R2 = 0.9892) (Additional file 2A). Melting curve analysis permitted the clear identification of *Pk*DNA, as shown additional file 2B, and T_*m*_ values for each sample were highly reproducible during repeated melt curve runs. The quantity of *Pk*DNA in urine samples collected from the monkey before, during, and after administration of anti-malarial treatment was measured by qPCR (Figure 4). The initial anti-malarial treatment started from 10:00 am on day 7 post-infection when parasitaemia was 10.08%. The quantity of *Pk*DNA in urine increased around 50-fold (50.3 P units) 6 hours after initial administration (Figure 4A). Furthermore, when parasitaemia was 9.58% (at 10:30 on day 20), a second anti-malarial treatment course was initiated. The quantity of *Pk*DNA in urine increased around 10-fold (5.0 P units) 6 hours after administration (Figure 4B). Twenty-four hours from the start of treatment for recrudescence, the concentration of *Pk*DNA markedly increased by around 60-fold (297.0 P units), followed by a concurrent decrease with decreasing parasitaemia.

### Comparison between microscopy and nPCR

**Figure 5:**
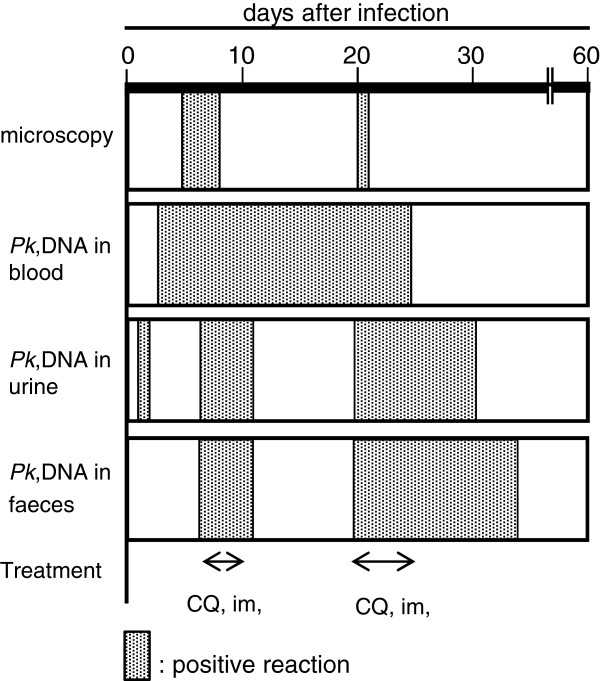
**Summary of parasite detection by microscopy and detection of** ***Pk*** **DNA by nPCR.** The arrows indicate intramuscular administration of chloroquine.

A summary of parasite detection by microscopy and of *Pk*DNA by nPCR performed on gDNA extracted from blood, urine and faeces is shown in Figure 5. The detection period of the parasite by microscopy was markedly shorter than the detection of *Pk*DNA using nPCR. *Pk*DNA in blood, urine and faeces was detectable by nPCR, even when the erythrocytic stage of the parasite was not observable by microscopy. Moreover, *Pk*DNA in faeces was detectable for a longer period than urinary *Pk*DNA and *Pk*DNA in the blood.

## Discussion

Diagnostic techniques based on PCR for the detection of *Plasmodium* DNA can be highly sensitive and specific. The vast majority of these techniques rely, however, on the invasive sampling of blood from infected hosts. Drawing blood for epidemiological surveys can cause considerable discomfort [2, 3]. Accordingly, follow-up of patients using a less-invasive approach is desirable when conducting surveys of malaria prevalence. Recent studies have reported the detection of *P. falciparum* DNA (*Pf*DNA) from saliva, urine and faecal samples, thus paving the way for a novel alternative source of specimens for potential malaria diagnosis [8–18]. These studies show that performing nPCR using *Pf*DNA templates extracted from noninvasive samples had encouragingly high sensitivity and specificity, compared with microscopy of infected blood. In the present study, we were able to monitor the presence of *Pk*DNA not only from blood samples, but also in urine and faeces were obtained from an infected Japanese macaque during a malaria episode.

Effective preservation of urine samples from malaria patients is a key factor influencing the performance of nPCR [11]. Furthermore, it has been suggested that ethanol preservation of urine samples is suitable for sample collection for field studies [11]. Although, in the present study, urine samples were not preserved in ethanol, they were promptly stored at −80°C, and the DNA extraction step was performed within a month of sampling. In addition, urine-derived DNA used as a PCR template was concentrated by centrifugal filter units prior to DNA extraction. A previous study using qPCR showed that the quantity of *Pf*DNA detected in human urine was 2500-fold less than that from blood obtained concurrently from infected individuals [9]. It is believed that trace amounts of parasite DNA in urine can be significantly affected by various external factors such as microbial contamination and temperature conditions during sample preservation. It therefore appears that using suitable preservation methods and carrying out a DNA concentration step are important for the detection of parasite DNA in urine.

The release of *Plasmodium* DNA in urine could be a general phenomenon that occurs during the course of infection. However, since *Plasmodium* DNA may be released into the urine via various possible routes, the actual route of entry has not yet been precisely defined. In the present study, urinary *Pk*DNA was first detected on day 2 after intravenous inoculation of frozen PRBCs, but was subsequently not detected on day 4, day 5 and day 6. The urinary *Pk*DNA seen on day 2 might have consisted of cell-free DNA from parasites that had degraded due to damage after thawing in the bloodstream. Moreover, qPCR results showed that the quantity of *Pk*DNA in the urine increased markedly following anti-malarial treatment. This might have been due to the release of a large amount of *Pk*DNA from the parasites that had been degraded following anti-malarial therapy, leading to excretion of *Pk*DNA in the urine. Therefore, the main source of *Plasmodium* DNA in urine is likely to consist of circulating cell-free DNA originating from dying parasites in peripheral blood. These findings indicate that urine sampling is potentially useful for detecting malaria cases in longitudinal surveillance during, for example, anti-malarial drug trials. Although the relationship between low parasitaemia and urinary *Pk*DNA level during chronic infection was not assessed here, the presence of *Pk*DNA in the urine may be explained by the passage of parasite DNA fragments through the glomerulus as a result of normal renal function. Indeed, the kinetics of both BUN and CREA levels in the host stayed within their normal range during the experiment.

The simian parasite *P. knowlesi* has recently been found to be a major cause of malaria in humans in Malaysian Borneo, with the disease also reported in several Southeast Asian countries [4]. Epidemiological surveys of *P. knowlesi* infections in wild monkeys are very important for assessing the risk of zoonotic malaria. However, few studies exist on zoonotic primate malaria occurring under natural conditions since it is practically and ethically difficult to obtain blood samples from wild monkeys [6, 7]. Faecal samples would, therefore, offer an attractive alternative method for the detection of primate malaria. Recently, phylogenetic analyses of DNA sequences from *Plasmodium* spp. have been conducted from faecal samples obtained from infected chimpanzees, gorillas, and bonobos [22–24]. The present study showed that *Pk*DNA was detectable by nPCR from faecal samples obtained over the course of an infection. Detection was possible, even when the parasite’s erythrocytic stage was not observable by microscopy. Our findings will provide basic data for future field surveys of *P. knowlesi* infection in free-living monkeys.

*Pk*DNA in the blood was detected from day 2 until day 25 post-infection. As DNA was extracted from whole blood containing RBCs and sera, DNA from both intra-erythrocytic parasites and parasite DNA fragments from the sera would be measured in this assay. *Pk*DNA was detected in the faeces over a longer period than urinary *Pk*DNA and the *Pk*DNA obtained from blood. Parasite DNA may enter the faeces passively via serum or within the phagosomes of macrophages in the host’s reticuloendothelial system. Another possibility is that cell-free DNA from degraded parasites in the liver enters the faeces via the bile. Recently, Abkallo and colleagues reported that DNA from the pre-erythrocytic stages of rodent malaria parasites was detectable in the liver, gall bladder and faeces of mice following sporozoite inoculation [25]. A particularly high concentration of parasite DNA was detected in the gall bladder. The authors concluded that parasite DNA entered the faeces via the bile following its clearance in the liver. Generally, infected erythrocytes are degraded not only in the spleen but also in the liver during the course of infection, and a large amount of degraded parasite constituents, including DNA, are produced in the liver. The degraded constituents are excreted from the liver via the bile to the faeces over a longer period compared to urine. It is possible that the parasite DNA is accumulated in gall bladder following parasite clearance, and it may be gradually excreted via the bile to the faeces. Whereas, the parasite DNA fragment through the glomerulus will not remain for very long in the urinary bladder, and it may be promptly excreted via the urine.

To date, molecular detection approaches using urine and faeces samples have been developed targeting several genes such as the merozoite surface protein-1 (*MSP1*) gene, *MSP2* gene, *DHFR* gene, small subunit ribosomal RNA gene (*18S rRNA*) and the mitochondrial cytochrome *b* (*cytb*) gene [8–13]. Putaporntip and colleagues demonstrated that the diagnostic performance of the *cytb*-PCR system using saliva and urine could be of practical value in comparison with performing *18S rRNA* gene-PCR [12]. It is thought that the *cytb*-PCR system is highly sensitive, because it targets the mitochondrial genome, multiple copies of which are present in each parasite. Furthermore, Mharakurwa and colleagues showed that shorter PCR amplicons are amplified effectively from parasite DNA in urine and saliva because such DNA may be highly fragmented by the time it reaches these fluids [8]. This result has previously also been observed with amplified human DNA derived from frozen urine samples [26]. In the present study, a 131-bp sequence of the *cytb* gene of *P. knowlesi* could be detected in urine and faeces using a specific PCR assay. To further develop the PCR system for detection of parasite DNA in excreta, it is necessary to consider the target gene as well as its PCR amplicon size.

## Conclusion

In conclusion, the *cytb*-PCR system using urine and faecal samples is of potential use in molecular epidemiological surveys of malaria. Caution must be taken, however, when interpreting the results of positive amplification of malaria parasite DNA from faeces, as Abkallo *et al*. demonstrated that this could derive from liver stage parasites, and so does not necessarily prove that a host has, or is even susceptible to, a blood infection with these parasites [25]. Bearing this in mind, faecal samples could be useful for the detection of zoonotic primate malaria in its natural hosts. These findings, therefore, emphasize the potential usefulness of the *cytb*-PCR system using urine and faeces as a tool for the surveillance of *P. knowlesi* infection in the field.

## Electronic supplementary material

Additional file 1: **A sampling net and tray were set under the monkey cage.** (PDF 114 KB)

Additional file 2: **The standard curve of qPCR for** ***Pk*** **DNA showing five** **10-fold** **serial dilutions (A).** The melt curve from qPCR for *Pk*DNA in urine samples **(B)**. (PDF 101 KB)

## Abbreviations

*Pk*DNA: *P. knowlesi* DNA
*Pf*DNA: *P. falciparum* DNA
*Cytb*: Cytochrome *b*.
