# Supplementary figures and images for: Detection of Plasmodium knowlesi DNA in the urine and faeces of a Japanese macaque (Macaca fuscata) over the course of an experimentally induced infection
Source: Malar J. 2014 Sep 19;13:373. doi: 10.1186/1475-2875-13-373 (PMC4177170; doi:10.1186/1475-2875-13-373)

Additional file 1

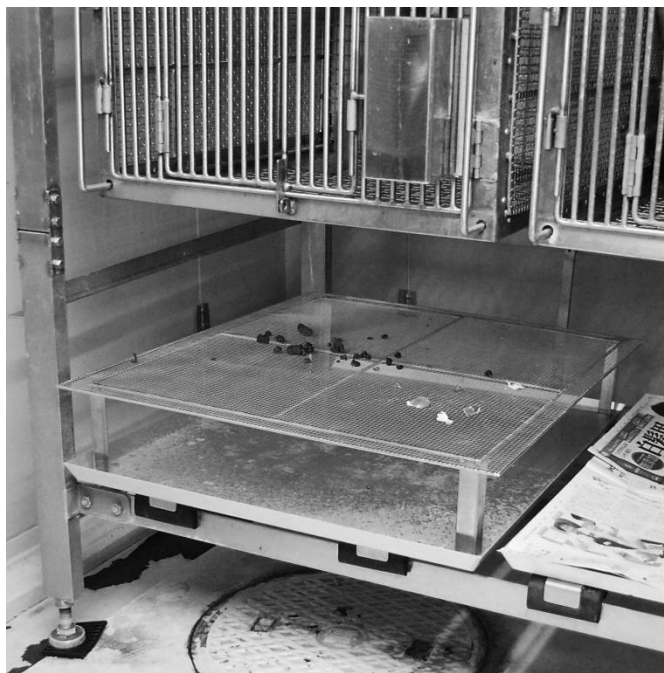

Supplement: Supplementary file 1 — Additional file 1: A sampling net and tray were set under the monkey cage. (PDF 114 KB) [file 12936_2014_3403_MOESM1_ESM.pdf]

## Additional file 2

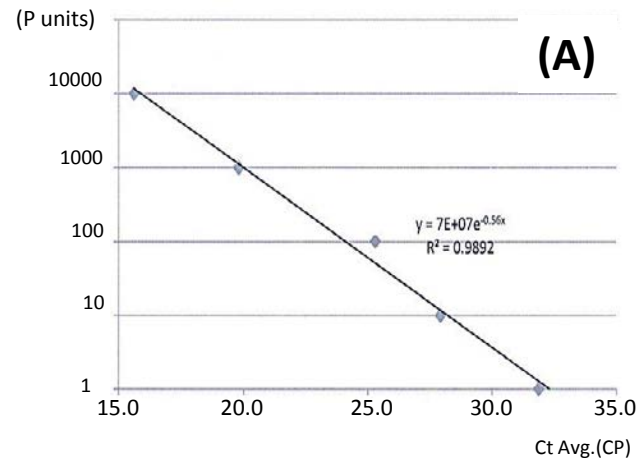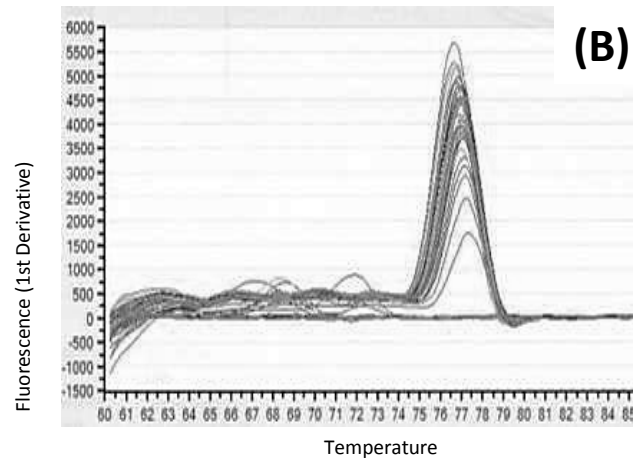

Supplement: Supplementary file 2 — Additional file 2: The standard curve of qPCR for Pk DNA showing five 10-fold serial dilutions (A). The melt curve from qPCR for PkDNA in urine samples (B). (PDF 101 KB) [file 12936_2014_3403_MOESM2_ESM.pdf]
